# Transgenesis-Mediated Reproductive Dysfunction and Tumorigenesis: Effects of Immunological Neutralization

**DOI:** 10.1371/journal.pone.0051125

**Published:** 2012-11-30

**Authors:** Ruchi Sachdeva, Neetu Bhardwaj, Ilpo Huhtaniemi, Usha Aggrawal, Swatantra Kumar Jain, Rana Zaidi, Om Singh, Rahul Pal

**Affiliations:** 1 Department of Biochemistry, Faculty of Science, Jamia Hamdard, New Delhi, India; 2 National Institute of Immunology, Aruna Asaf Ali Marg, New Delhi, India; 3 Department of Physiology, University of Turku, Turku, Finland; 4 Department of Reproductive and Developmental Biology, Imperial College, London, Hammersmith Campus, London, United Kingdom; 5 Institute of Pathology, Safdarjung Hospital, New Delhi, India; Medical Faculty, Otto-von-Guericke University Magdeburg, Medical Faculty, Germany

## Abstract

Human chorionic gonadotropin (hCG) was initially thought to be made only during pregnancy, but is now known to also be synthesized by a variety of cancers and is associated with poor patient prognosis. Transgenic expression of βhCG in mice causes hyper-luteinized ovaries, a loss in estrous cyclicity and infertility, increased body weight, prolactinomas and mammary gland tumors. Strategies were devised to generate antibody responses against hCG to investigate whether reversal of the molecular processes driving tumorigenesis would follow. hCG-immunized transgenic mice did not exhibit increases in body weight or serum prolactin levels, and gross ovarian and pituitary morphology remained normal. While non-immunized transgenic animals demonstrated heightened levels of transcripts associated with pituitary tumorigenesis (HMG2A, E2F1, CCND1, PRL, GH, GAL, PTTG1, BMP4) and decreased levels of CDK inhibitors CDKN1B (p27), CDKN2A (p16) and CDKN2c (p18), immunization led to a reversal to levels found in non-transgenic animals. Serum derived from transgenic (but not non-transgenic) mice led to enhanced transcription as well as expression of VEGF, IL-8, KC (murine IL-8) and MMP-9 in tumor cells, effects not seen when sera derived from hCG-immunized transgenic mice was employed. As the definitive indication of the restoration of the reproductive axis, immunization led to the resumption of estrous cyclicity as well as fertility in transgenic mice. These results indicate that hCG may influence cancer pathogenesis and progression via several distinct mechanisms. Using a stringent *in vivo* system in which βhCG acts both a “self” antigen and a tumor-promoting moiety (putatively akin to the situation in humans), the data builds a case for anti-gonadotropin vaccination strategies in the treatment of gonadotropin-dependent or secreting malignancies that frequently acquire resistance to conventional therapy.

## Introduction

Human chorionic gonadotropin (hCG) is a heterodimeric glycoprotein hormone produced by placental trophoblasts during pregnancy. Only hCG α-β dimer is considered biologically active and sustains ovarian steroidogenesis. hCG is also ectopically expressed by a wide variety of trophoblastic and non-trophoblastic cancers. Its presence has been associated with poor prognosis in variety of cancers [1]–[3], with some evidence of association with chemo-resistance [4], [5].

Newly-developed animal models lend further weight to the postulate linking hCG with tumorigenesis. For example, female transgenic mice expressing βhCG under the ubiquitin C promoter develop precocious puberty, disrupted estrous cycles and infertility due to massive luteinization in the ovaries; animals develop obesity, pituitary prolactinomas and mammary gland adenocarcinomas [6], [7]. Extra-gonadal phenotypic changes are abolished by gonadectomy. A recent report suggests a role for progesterone in the growth of pituitary adenomas via concomitant activation of oncogenes HMGA2 and E2F1 and the downregulation of the retinoblastoma (RB) protein [8].

Given the postulated and established tumor-promoting roles of hCG, targeting the molecule may prove to be a viable immunotherapeutic strategy. A role for both hCG-specific cytotoxic T cells and antibodies can be envisaged. Previous work in our lab has shown that it is indeed possible to break tolerance and induce bioeffective antibody responses towards βhCG in humans by carrier conjugation [9] and vaccination of colorectal cancer patients with the carboxy-terminal peptide (CTP) of βhCG coupled to diphtheria toxoid has been shown to have beneficial effects on survival [10].

The need of the hour is to develop suitable animals models in which anti-hCG vaccination strategies can be tested in physiological conditions akin to humans. In the present study, the effects of a variety of anti-hCG vaccine formulations were assessed in βhCG transgenic (TG) female mice. hCG was immunized along with Complete and Incomplete Freund’s Adjuvant. Additionally, immunizations were also carried out with an alum-adsorbed βhCG-tetanus toxoid (TT) conjugate, with and without supplementation with *Mycobacterium indicus pranii* (MIP), with the expectation that the bacterium would provide additional adjuvantic effects.

In view of the demonstrated growth-promoting effects of βhCG on tumor cells [11]–[13], sera derived from TG mice, wild-type (WT) mice as well as from immunized TG mice were assessed for effects on tumor cell viability. Since emerging evidence appears to suggest that hCG may also exhibit angiogenic [14] and pro-invasive [15] activity, sera from non-immunized and immunized TG mice were evaluated for the ability to induce the transcription and expression of vascular endothelial growth factor (VEGF), KC (murine IL-8) as well as of matrix mellatoprotease (MMP)-9 from tumor cells. Effects of immunization on body weight were assessed and serum prolactin levels estimated. Histological examination of the ovaries and pituitaries was carried out to determine whether effective anti-hCG immunization has ameliorating effects. The effect of immunization on genes known to be associated with pituitary tumorigenesis, including those in the CCND/CDK1/RB/E2F1 pathway, was also assessed. Lastly, whether such immunization negated disruptions in estrous cyclicity and fertility observed in TG animals was also evaluated. These studies provide further mechanistic insights into a specific instance of hCG-induced tumorigenesis and unequivocally establish the efficacy of an anti-hCG vaccination strategy, using an appropriate “self” animal model in which βhCG/hCG acts as an endogenous tumor promoter.

## Materials and Methods

### Ethics Statement

This study was carried out in strict accordance with the protocol approved by the Institutional Animal Ethics Committee (IAEC) of the National Institute of Immunology, New Delhi (IAEC Number: 231/10). Blood samples were withdrawn under ketamine and xylezine anaesthesia and all efforts were made to minimize suffering.

### βhCG Transgenic Mice

The generation of ubiquitin C/βhCG TG mice has been described previously [6]; heterozygous transgenic males were bred with wild-type FVB/N females.

### Hormone Levels

Serum βhCG in TG female mice was estimated by radioimmunoassay (RIA). Briefly, diluted sera (or varying concentrations of an hCG standard) were incubated with ^125^I-hCG, 20% normal horse serum (NHS) and an appropriately diluted monoclonal antibody against βhCG at 4°C for 24 hr. The antigen-antibody complex was precipitated by the addition of 12.5% (w/v) polyethylene glycol (PEG) (Sigma Aldrich) followed by centrifugation at 1800 g at 4°C for 30 min. Radioactivity in the pellet was assessed in a gamma counter (Perkin Elmer) and serum βhCG concentrations were estimated with reference to the hCG standards. Serum prolactin levels were estimated by ELISA (R&D Systems).

### Genomic PCR for βhCG

Genomic DNA was denatured at 94°C for 5 min. PCR was performed using primers specific for the ubiquitin promoter (5′-CGCGCCCTCGTCGTGTC-3′) and βhCG (5′-AAGCGGGGGTCATCACAGGTC-3′) as previously described [6].

### Immunization

The conjugation of βhCG with TT was carried out as previously described [16]. The βhCG-TT conjugate was adsorbed on Alhydrogel (Superfos, Denmark) prior to immunization. MIP was grown in Middlebrook 7H9 media (BD DIFCO) supplemented with 10% albumin-dextrose complex enrichment (BD DIFCO), 0.02% glycerol, and 0.05% Tween 80. Mycobacteria were harvested by centrifugation at 840 g for 15 minutes and washed thrice with PBS. Twelve-week old TG and WT mice (n = 12) were immunized with three fortnightly injections of 2 µg βhCG equivalent of the conjugate with or with additional supplementation with 10^7^ autoclaved MIP. In the third experimental set, mice were immunized subcutaneously with 10 µg hCG emulsified in Complete Freund’s Adjuvant for first injection followed by two fortnightly injections at the same dose in Incomplete Freund’s Adjuvant. In the fourth experimental set, mice were immunized with three fortnightly injections of 10 µg hCG emulsified in Incomplete Freund’s Adjuvant.

### Anti-hCG Antibody Titres

Serum anti-hCG antibody titres were estimated by RIA essentially as described [16]. Briefly, diluted sera from immunized TG and WT mice were incubated with ^125^I-hCG and 20% NHS at 4°C for 36 hr. Antigen-antibody complexes were precipitated by the addition of 12.5% (w/v) PEG and centrifugation at 1800 g at 4°C for 30 min. At appropriate dilutions of sera, results were expressed as percentage of added ^125^I-hCG in the bound fraction. Bioneutralization capacity of the elicited antibodies was measured as previously described [17]. Results were expressed as percentage inhibition in the binding of ^125^I-hCG towards rat testicular receptors.

### Cell Viability Analysis

5 x 10^4^ COLO 205 (human colorectal carcinoma), ChaGo (human lung carcinoma) or Lewis lung carcinoma (LLC; murine lung carcinoma) cells were obtained from ATCC and cultured in serum-free medium (BioWhittaker), or serum-free medium containing either of the following: serum derived from TG mice, WT mice, immunized TG mice, or from immunized WT mice. Cells were also cultured with serum derived from TG animals plus heat inactivated anti-hCG serum or normal serum. After incubation for 24 hr, MTT (3-[4, 5-dimethylthiazol-2-yl]-2,5-diphenyltetrazolium bromide; Sigma) was added, followed by an incubation for 5 hr. After addition of the stop solution (50% DMSO in 20% SDS), absorbance was recorded at 550 nm.

### Transcription and Expression of VEGF, IL-8 and MMP-9

5×10^5^ COLO 205, ChaGo or LLC cells were incubated with serum derived from either TG mice, WT mice, immunized TG mice, or immunized WT mice for 24 hr. Cells were also co-incubated with serum derived from TG animals along with heat inactivated anti-hCG serum or normal serum. Levels of VEGF (Peprotech), IL-8 (Peprotech) and KC (murine IL-8; R&D systems) were quantified in culture supernatants by ELISA.

Cell supernatants were electrophoresed on a 10% SDS-polyacrylamide gel containing 0.2% gelatine (Sigma). Gels were washed with 2.5% Triton-X-100, followed by incubation in 0.05 mM Tris–HCl buffer, pH 8.8 (containing 5 M CaCl_2,_ and 0.02% sodium azide) for 24 hr at room temperature. Gels were stained with Coomassie Brilliant Blue R250 (Gibco BRL) to visualize enzymatic (gelatinolytic) activity.

1 µg total RNA, obtained from cells subsequent to the experiment described above, was reverse transcribed into cDNA using oligo dT and reverse transcriptase (Promega). Semi-quantitative PCRs were carried out using primers specific for VEGF, IL8 and MMP-9; primer sequences are listed in Table S1. Samples were subjected to a 15 min denaturation step at 94°C, followed by 40 cycles of three steps each: 94°C for 1 min, annealing for 1 min, extension at 72°C for 1 min, followed by final extension at 72°C for 10 min.

### Reverse Transcriptase-PCR for Transcripts of Proteins Implicated in Pituitary Tumorigenesis

Total RNA was isolated from the pituitaries of 10-month old (immunized and control) TG and WT female mice.1 µg of RNA was reverse transcribed into cDNA (Promega). PCRs were performed using primers specific for factors implicated in pituitary tumorigenesis (Table S2). Denaturation was carried out at 94°C for 1 min, followed by annealing for 1 min, extension at 72°C for 1 min and final extension at 72° for 10 min.

### Histological Analysis

Ovaries and pituitary glands were isolated from TG (control and immunized) mice. Tissues were fixed in 10% formaldehyde and embedded in paraffin. 5 µm sections were stained with hematoxylin and eosin and images were captured on a digital camera (Canon).

### Estrous Cyclicity and Fertility

Estrous cyclicity was assessed in TG (control and immunized) female mice and in WT mice by vaginal smear cytology for a minimum of 3 consecutive cycles. For determination of fertility status, control and immunized transgenic females were mated with wild-type FVB/N male mice, and incidence of pregnancy and number of live births were recorded.

### Statistical Analysis

Statistical analysis was carried out using the Student’s t-test.

## Results

### Characterization of βhCG Transgenic Mice

**Figure 1:**
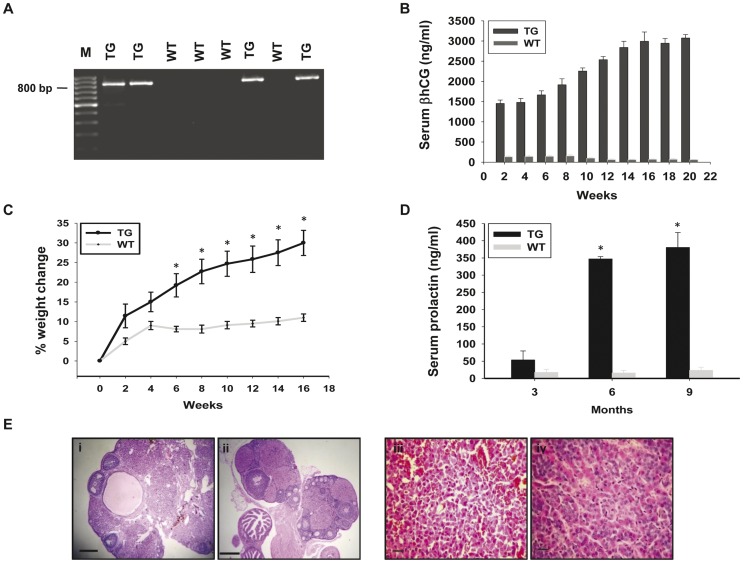
Characterization of βhCG transgenic mice. (A) Genomic PCR for the βhCG transgene, using DNA derived from the peripheral blood cells of F1 mice (see text for details). A PCR product (∼800 bp) was observed in about 50% of the animals, as expected. TG: Transgenic mice; WT: Wild-type mice. M: 100 bp ladder. (B) Serum βhCG levels in TG and WT mice (n = 10) as a function of age. Arithmetic means±standard errors are shown. (C) Percentage change in body weight in TG and WT mice (n = 10) as a function of age. Arithmetic means±standard errors are shown. *p<0.05 v/s corresponding weight change in wild-type mice. (D) Serum prolactin levels in TG and WT mice (n = 10) as a function of age. Arithmetic means±standard errors are shown. *p<0.05 v/s respective prolactin levels in wild-type mice. (E) Hemotoxylin-eosin stained histological sections of the (i, ii) ovaries (Bars: 100 µm) and (iii, iv) pituitaries (Bars: 20 µm) of (i, iii) TG and (ii, iv) WT female mice at 10 months of age.

The βhCG TG animals employed in this study have been previously described [6]. PCR, performed on genomic DNA from PBMCs isolated from F1 progeny, revealed the presence of the transgene in about half the individuals, as expected (Figure 1A). Serum βhCG and prolactin levels demonstrated age-related increases in TG animals (Figure 1B, 1D). TG female mice steadily gained weight in comparison with wild-type females due to the deposition of fat in the abdominal cavity (Figure 1C). Extensive luteinization was evident in ovaries obtained from 10-month old TG mice. Multiple corpora lutea and hemorrhagic cysts were observed, with the few remaining follicles pushed to the periphery (Figure 1E (i, ii)). Histological analysis of pituitary glands derived from TG animals was indicative of hyperplasia; by 12 months, pituitary size increased significantly (data not shown) and adenomas were apparent. In contrast to pituitaries derived from wild-type mice which demonstrated a mixed population of acidophilic and basophilic cells, pituitaries from TG mice predominantly contained basophilic cells with enlarged nuclear and cytoplasmic inclusions and abundant cytoplasm (Figure 1E (iii, iv)). Estrous cyclicity, whilst normal in wild-type females, was disrupted in TG females (data not shown), who were also infertile.

### Effects of Immunization on Antibody Titres, Weight Gain and Serum Prolactin

**Figure 2:**
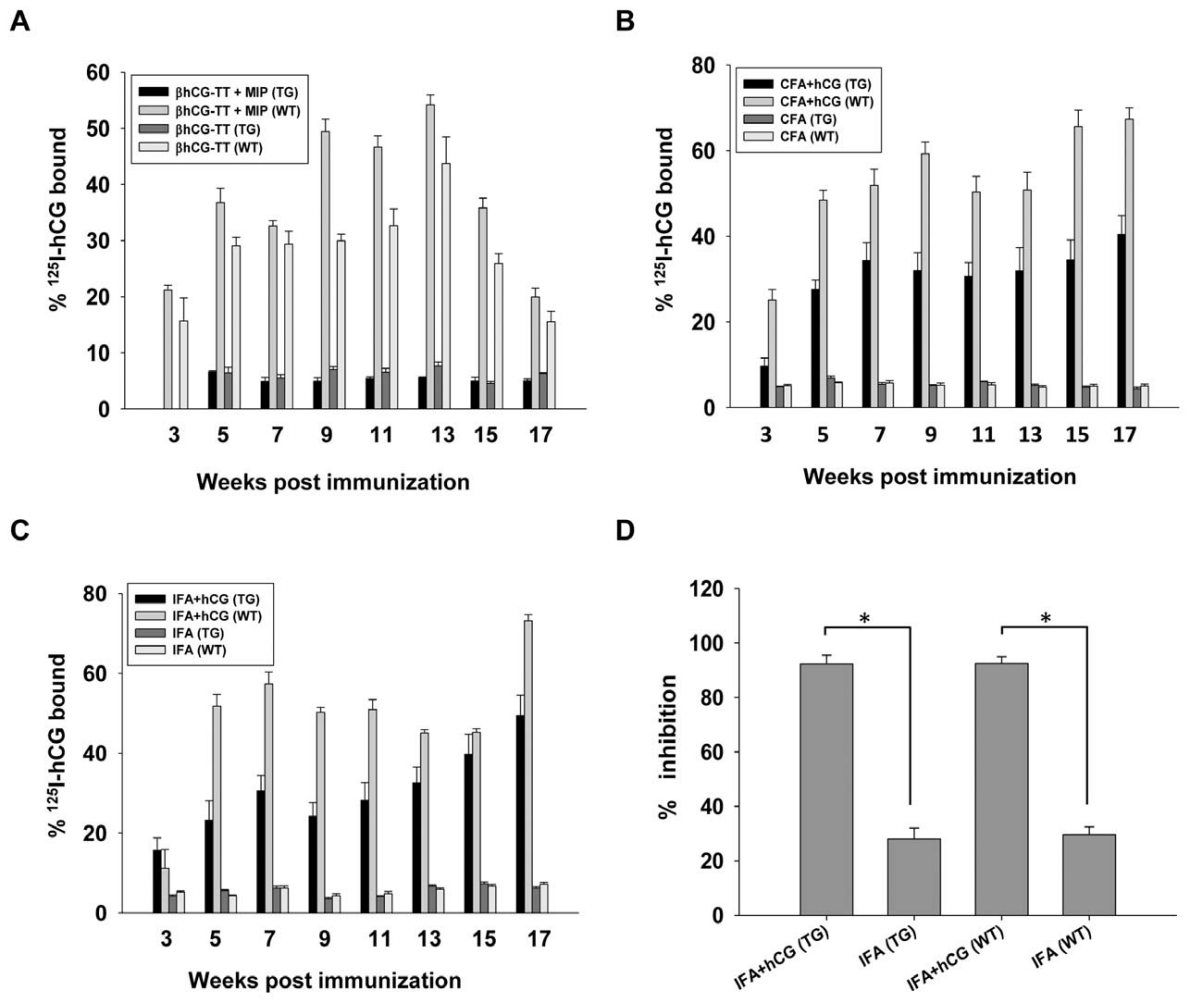
Characterization of anti-hCG antibody responses in TG and WT mice. (A) Serum anti-hCG antibody titres in TG (n = 12) and WT (n = 12) mice immunized with either βhCG-TT or βhCG-TT + *MIP*, as a function of time. (B) Serum anti-hCG antibody titres in TG (n = 12) and WT (n = 12) mice immunized with CFA or CFA + hCG, as a function of time. (C) Serum anti-hCG antibody titres in TG (n = 12) and WT (n = 12) mice immunized with IFA or IFA + hCG, as a function of time. (D) Estimation of the capacity of antibodies in the sera of TG (n = 12) and WT (n = 12) mice (immunized either with IFA or IFA + hCG) to inhibit hCG-receptor interaction. In all cases, arithmetic means±standard errors are shown. *p<0.01.

Immunization of WT female mice with a βhCG-TT conjugate adsorbed on alum (a formulation previously shown to be immunogenic in humans [9], [16]), with or without additional supplementation with MIP, resulted in significant anti-hCG antibody responses; immunization of TG female mice with similar formulations did not lead to the generation of measurable serum anti-hCG antibody titres (Figure 2A). Immunization of both WT and TG female mice with hCG emulsified in either CFA or IFA, on the other hand, resulted in the generation of anti-hCG antibodies (Figure 2B, 2C); antibodies were capable of inhibiting hCG-receptor interaction (Figure 2D).

**Figure 3:**
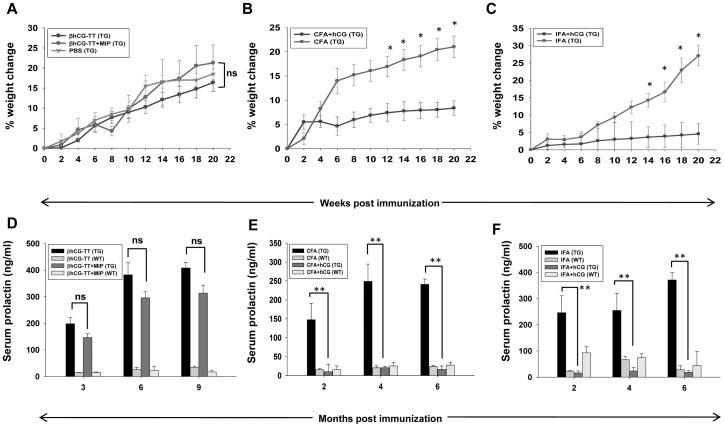
Effects of active immunization against hCG on weight gain and serum prolactin levels. (A) Percentage weight change and (D) serum prolactin levels in TG (n = 12) and WT (n = 12) mice immunized with either βhCG-TT or βhCG-TT + *MIP*, as a function of time. (B) Percentage weight change and (E) serum prolactin levels in TG (n = 12) and WT (n = 12) mice immunized with CFA or CFA + hCG, as a function of time. (C) Percentage weight change and (F) serum prolactin levels in TG (n = 12) and WT (n = 12) mice immunized with IFA or IFA + hCG as a function of time. *p<0.05 v/s corresponding weight change in transgenic mice immunized with adjuvant + hCG; **p<0.01; ns = not significant.

Immunization of TG mice with either βhCG-TT or βhCG-TT + MIP did not alter the rate of weight gain compared with that observed in non-immunized TG animals (Figure 3A); serum prolactin levels in these animals remained significantly elevated over those observed in WT mice (Figure 3D). Both CFA and IFA-based anti-hCG immunization caused significant reduction in the rate of weight gain in TG animals, compared with animals immunized only with adjuvant (Figure 3B, 3C), as well as caused a reduction of serum prolactin to levels observed in wild-type mice (Figure 3E, 3F).

### Effects of Immunization on Potential Pro-tumorigenic Effects of TG Serum

**Figure 4:**
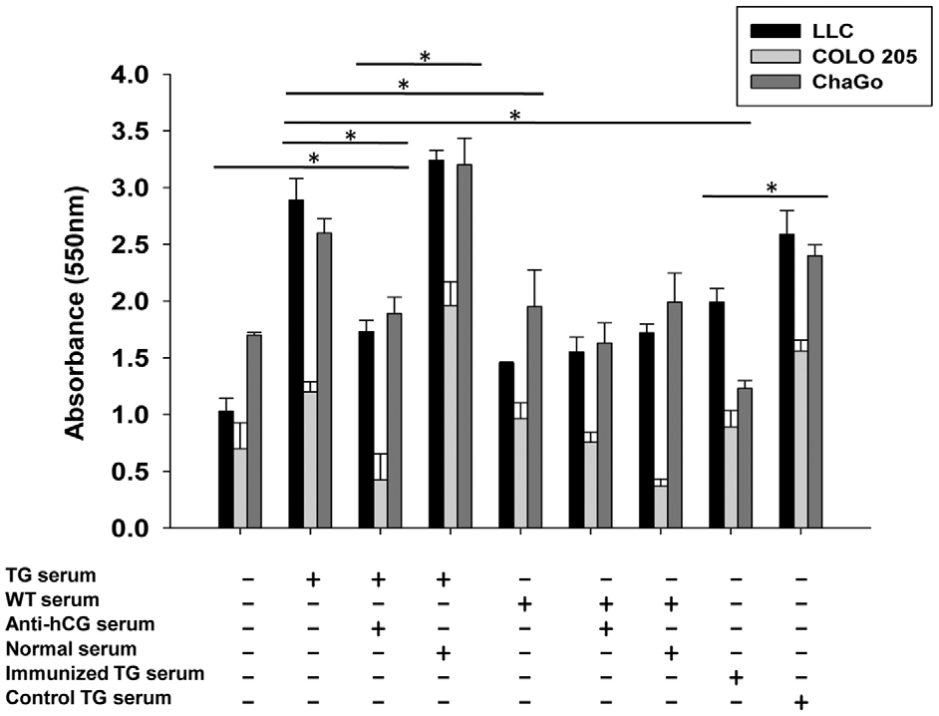
Effects of TG serum on cell viability. Effect of serum (1∶4) derived from TG mice (“TG serum”), from TG mice immunized with hCG + IFA (“Immunized TG serum”), from TG mice immunized with IFA (“Control TG serum”) and from WT mice on the viability of LLC, COLO 205 and ChaGo cells was assessed by MTT analysis. The effects of co-incubation of TG serum with exogenous anti-hCG serum (1∶200), or with normal serum (1∶200), are also shown. The horizontal lines in the figure extend across the bars which have been compared, at their two ends. In each case, the asterisk indicates significance (*p<0.05) for all three cell lines being compared.

On the basis of previous reports [11]–[13] as well as unpublished data on the growth-promoting effects of βhCG on tumor cells, whether serum derived from TG animals could promote the growth of tumor cells was ascertained. LLC, COLO 205 and ChaGO cells demonstrated enhanced viability when incubated with TG serum but not with serum obtained from WT mice. Pre-incubation of TG serum with exogenous anti-hCG antiserum (but not with normal serum) abolished effects on cell viability. Additionally, tumor cells incubated with serum derived from TG mice immunized with hCG plus adjuvant did not experience enhanced viability, whereas tumor cells incubated with serum derived from TG mice immunized with adjuvant alone did (Figure 4).

**Figure 5:**
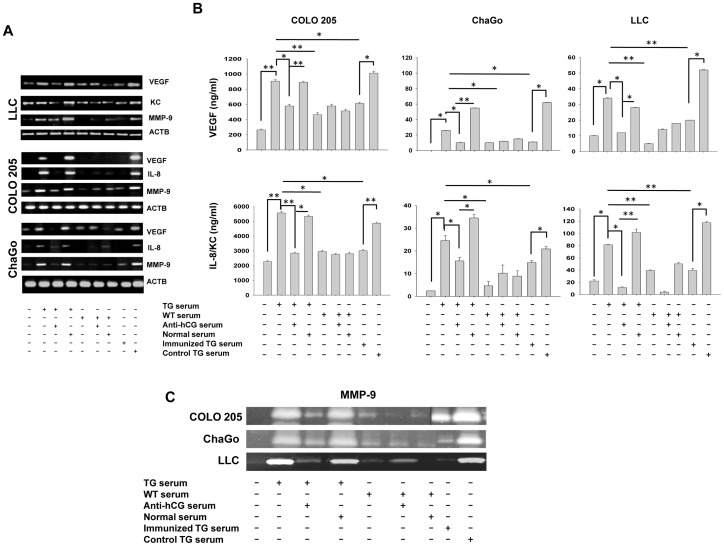
Tumor promoting effects of TG serum. (A) Semi-quantitative RT-PCR analysis of VEGF, IL-8 (or KC) and MMP-9 transcript levels in LLC, COLO 205 and ChaGo cells. ACTB (β-actin) served as control. Incubation conditions were as described in Figure 4. (B) VEGF, IL-8 (or KC) concentrations in culture supernatants of LLC, COLO 205 and ChaGo cells. Incubation conditions were as described in Figure 4. *p<0.05; **p<0.01 between the conditions indicated. (C) Zymography analysis demonstrating gelatinolytic activity of culture supernatants obtained from LLC, COLO 205 and ChaGo cells. Incubation conditions were as described in Figure 4.

The effect of sera derived from TG and WT mice on the transcription and expression of VEGF, IL-8 and MMP-9 in tumor cells was then assessed, since these molecules play key roles in the process of tumorigenesis and metastasis [18]–[24]. In all instances, PCR analysis revealed that TG sera enhanced transcript levels over those observed when tumor cells were incubated with sera derived from WT mice. Pre-incubation of TG sera with anti-hCG antiserum (but not with normal serum) abolished these enhancements. Further, sera derived from TG mice which had been immunized with hCG plus adjuvant, as opposed to sera derived from TG mice immunized with adjuvant alone, was also unable to induce such enhancements (Figure 5A). ELISAs (for VEGF and IL-8) and zymogram analysis (for MMP-9) by and large revealed a correlation between expressed protein and transcript levels under each of the conditions outlined above (Figures 5B, 5C).

### Effect of Immunization on Tumor-associated Pituitary Transcripts in TG Mice

**Figure 6:**
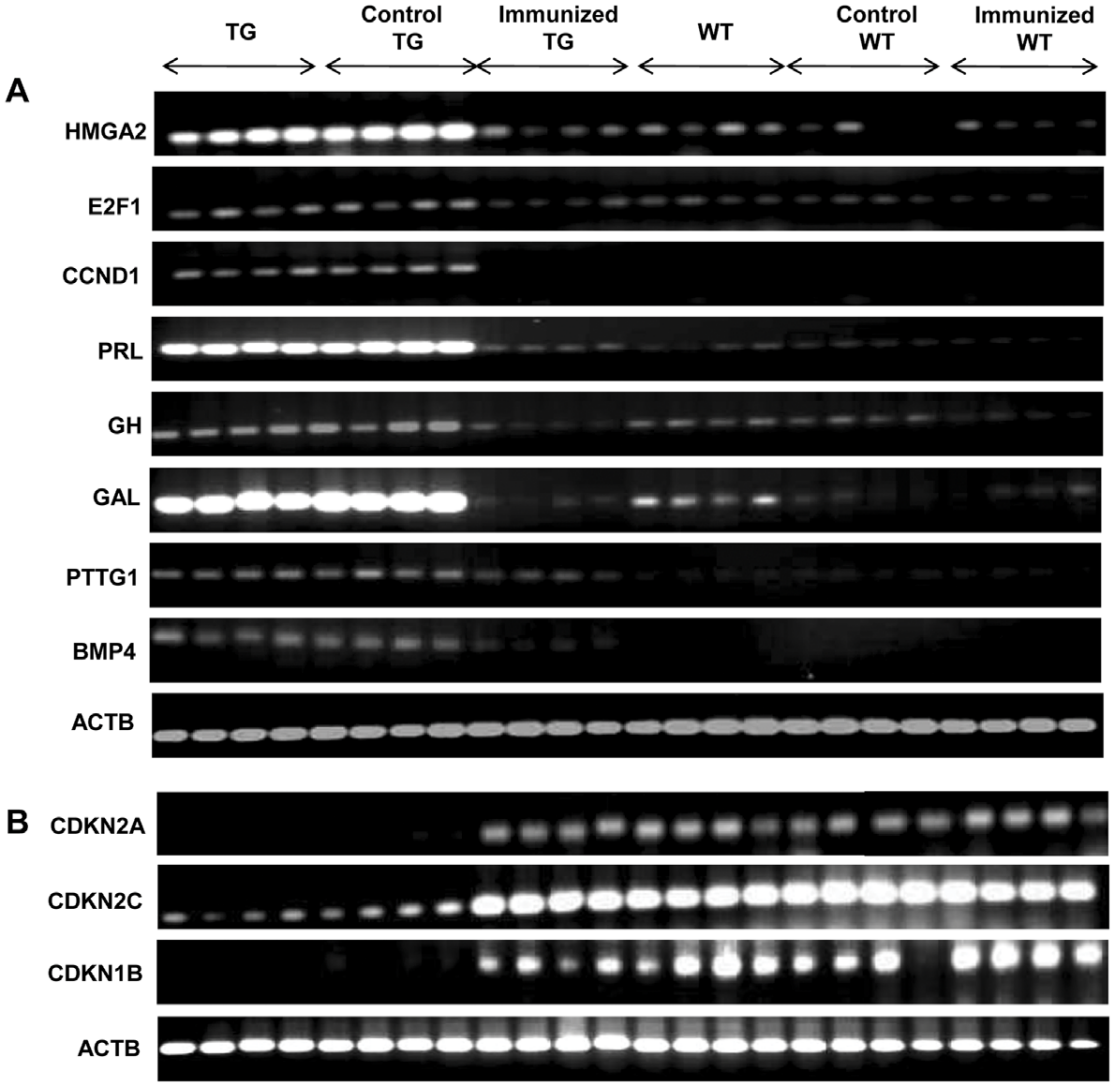
Effects of active immunization against hCG on tumor-associated pituitary transcripts. (A) Semi-quantitative RT-PCR analysis of transcripts of tumor-associated proteins (HMGA2, E2F1 CCND1, PRL, GH, GAL, PTTG1, BMP4) and (B) CDK inhibitors (CDKN1B, CDKN2A and CDKN2C) in the pituitaries derived from TG mice, TG mice immunized with IFA (“Control TG”), TG mice immunized with hCG + IFA (“Immunized TG”), WT mice, WT mice immunized with IFA (“Control WT”) and WT immunized with hCG + IFA (“Immunized WT”). Each lane represents an individual animal. ACTB (β-actin) served as control.

Pituitaries derived from control and immunized TG and WT mice were evaluated for relative transcript levels of a number of genes known to be associated with the development of prolactinomas [8], [25]–[33]. Increased transcription of HMGA2 (high mobility group AT-hook2), E2F1 (E2F transcription factor 1) and CCND1 (cyclin D1), PRL (prolactin) and GH (growth hormone) was observed in the pituitaries of TG mice in comparison with WT animals. Similarly, transcript levels of GAL (galanin), a marker for lactotrophs [28], was highly expressed in the pituitaries of TG mice, as were transcripts for BMP4 (bone-morphogenetic protein 4, a signalling molecule required for early embryonic and pituitary development [29]) and PTTG1 (pituitary tumor transforming growth factor 1, an oncogene expressed in pituitary adenomas [30], [31]). While immunization of TG mice with hCG plus adjuvant decreased transcript levels of these genes to those observed in wild-type levels, immunization with adjuvant alone had no effect (Figure 6A). CDK inhibitors CDKN1B (p27), CDKN2A (p16), and CDKN2C (p18) are involved in G1-S transition, provide growth inhibitory signals and exhibit alterations in pituitary adenomas [32], [33]. Pituitaries derived from TG mice demonstrated reduced transcript levels of these genes compared with WT mice. Immunization of TG mice with hCG plus adjuvant (but not with adjuvant alone) enhanced transcript levels of all three inhibitors to WT levels (Figure 6B).

### Effects of Immunization on Ovarian and Pituitary Histology

**Figure 7:**
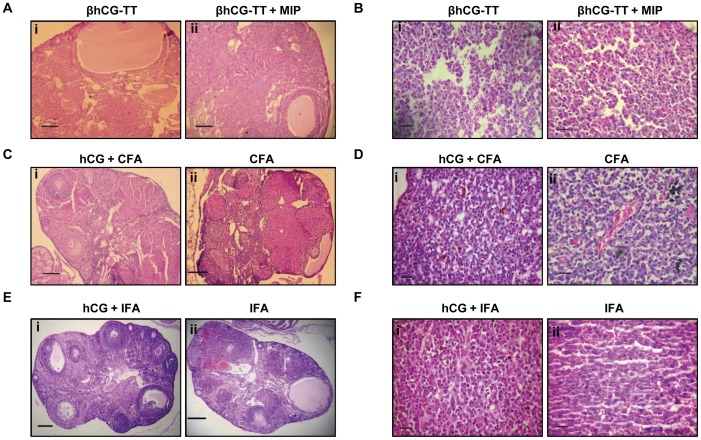
Effects of active immunization against hCG on ovarian and pituitary histology. Histological analysis of (A, C, E) the ovaries (Bars: 100 µm) and (B, D, F) the pituitaries (Bars: 20 µm) upon immunization of TG animals with (A(i), B(i)) βhCG-TT, (A(ii), B(ii)) βhCG-TT + *MIP*, (C(i), D(i)) hCG + CFA, (C(ii), D(ii)) CFA, ((E(i), F(i)) hCG + IFA or ((E(ii), F(ii)) IFA.

Immunization of TG mice with βhCG-TT or βhCG-TT+MIP did not result in significant histological changes in the organs; as in non-immunized TG animals, the ovaries exhibited extensive luteinization and the presence of cysts, and the pituitaries demonstrated hyperplastic and adenomatous changes (Figure 7A, 7B). On the other hand, TG mice immunized with either the CFA or IFA-based formulations (as opposed to mice immunized with adjuvant alone) demonstrated normal ovarian morphology; follicles at different stages of development were observed, as were fewer corpora lutea and a total absence of cysts (Figure 7C, 7E). Pituitary glands were morphologically normal with a mixed population of acidophilic, basophilic and chromophobic cells (Figure 7D, 7F). Immunization of WT mice with any of the three formulations did not induce observable changes in ovarian or pituitary morphology (data not shown).

### Fertility Studies

The fact that immunization of TG mice with hCG plus adjuvant led to restorative changes in the ovaries and pituitaries, accompanied by an associated decline in serum prolactin levels, prompted evaluation of fertility status. Anti-hCG immunization resulted in a return to estrous cyclicity in six out of seven animals; immunization with adjuvant alone, on the other hand, had no effect on cyclicity (data not shown). Upon mating with wild-type FVB/N males, while none of the non-immunized TG females conceived, one out of seven TG females immunized with adjuvant alone conceived and delivered a litter of three pups. In contrast, five of seven TG animals immunized with hCG plus adjuvant conceived, resulting in a total of forty five pups. Conception rates and litter size in WT animals immunized either with hCG plus adjuvant or adjuvant alone remained unaffected, in comparison with non-immunized WT animals (Table 1).

**Table 1:** Effects of active immunization against hCG on fertility.

Groups	n	Number pregnant	Litter size
TG	7	0	0
WT	7	7	56
Immunized TG	7	5	45
Control TG	7	1	3
Immunized WT	7	7	56
Control WT	7	7	58

Female mice (non-immunized (“TG and WT”), immunized with hCG + IFA (“Immunized TG” and “Immunized WT”) or immunized with IFA (“Control TG” and “Control WT”)) were mated with WT FVB/N males. Incidence of pregnancy and litter size was recorded.

## Discussion

Gonadotropin levels are elevated in physiological states such as pregnancy and menopause, and can also be heightened in pathological conditions. Epidemiological data has drawn correlation between lifetime exposure to gonadotropins and the occurrence of ovarian cancer [34]. hCG is now know to be made by variety of cancers of non-trophoblastic origin, for example those of the colon, prostate, bladder, breast and lung. βhCG secreted by tumor cells exhibits growth factor-like properties [11]–[13]. The presence of membrane-associated βhCG has been shown to correlate with the metastatic potential of tumor cells [35] and expression of the molecule is associated with poor prognosis [1]–[3]. A naturally-occurring activating LH receptor mutation (Asp578His) is associated with Leydig cell adenoma in humans [36]. Evidence from humans as well as from transgenic murine models have also provided evidence of a role for gonadotropins in tumorigenesis [6]–[8], [37], [38].

Neutralization of hCG has shown to be effective in regulating tumor cell growth both *in vivo* and *in vitro*. Significant decrease in hCG production and cell proliferation is observed in Jar choriocarcinoma cells transfected with antisense βhCG [39]. Anti-βhCG antiserum has been shown to reduce βhCG-induced proliferative responses in bladder cancer cells [11]. Administration of anti-hCG antibodies blocks tumor formation as well as growth *in vivo* [40], [41]. Immunization of mice with the carboxy-terminal peptide (CTP) of hCG (linked with mycobacterial heat shock-protein HSP65) has been shown to effectively inhibit the growth of Lewis Lung carcinoma [42]. Evidence for the beneficial effects of hCG neutralization has also been obtained in patients of colorectal cancer [10].

This study was designed to investigate the potential of active anti-hCG vaccination strategies in the control of tumorigenesis by the use of transgenic animals in which βhCG/hCG behaves both as a “self” antigen as well as a tumor-promoting moiety. As originally reported [6] and subsequently reproduced in this study, βhCG transgenic mice develop pituitary adenomas and hyperprolactinemia and, possibly as a direct consequence [43], a clearly obese phenotype. The pituitary tumours produced by the TG mice are pure prolactinomas [6], and they do not produce gonadotropins, TSH or ACTH. Ovarian hypertrophy and disturbances in estrous cyclicity are other notable physiological aberrances. The infertility of these mice appears to be due to the very high prolactin levels in young adults, because it can be reversed by temporary inhibition of prolactin secretion by cabergoline [44]. TG and WT mice were immunized with a variety of anti-hCG vaccine formulations. Interestingly, formulations comprising of βhCG-TT adsorbed on alum, while inducing high titre anti-hCG antibodies in WT animals, failed to induce such antibodies in TG animals. In extensive clinical trials by our group, βhCG-TT (as well as analogous carrier conjugates) have invariably demonstrated significant immunogenicity in human females [9], [16], [17] and have served as antigens for successful anti-fertility vaccine clinical trials [9], indicating the ability of such conjugates to break self-tolerance to hCG in humans. Further, while recent work in our lab has demonstrated that the inclusion of MIP in βhCG-TT based formulations significantly enhances titres of neutralizing anti-hCG antibodies in several murine strains (unpublished data), the current study found that MIP-containing βhCG-TT formulations were also non-immunogenic in TG animals. It may be speculated that, unlike human (non-pregnant) females, TG mice express appreciable levels of βhCG (≅ 1–5 µg/ml) in serum; levels of adjuvantic assistance provided by the alum-based formulations, while being sufficient in humans, may therefore be inadequate at eliciting demonstrable antibody responses in TG mice. In consonance with the lack of immunogenicity, immunization with the alum-based formulations did not result in significant effects on either the kinetics of increases in body weight or on serum prolactin levels. Immunization of TG mice with hCG emulsified in either CFA or IFA, on the other hand, resulted in the generation of appreciable titres of bio-effective anti-hCG antibodies; the kinetics of weight gain in these animals was akin to that observed in WT mice, serum prolactin levels declined to near-baseline levels and the pituitaries exhibited normal morphology. Since it is believed that prolactin may significantly contribute towards the maintenance of structural and functional integrity of the corpus luteum in rodents [45], decreased prolactin levels upon immunization, along with effective neutralization of βhCG/hCG, was probably responsible for the absence of pathological changes in ovaries recovered from TG animals immunized with hCG plus adjuvant.

E2F1 has been associated with the presence of pituitary adenomas in mice [25]. Further, CCND1/CKD4 and E2F1 have been previously reported to be up-regulated, and Rb1 down-modulated, in the adenomatous pituitary of the TG female mice employed in the current study [8]. Heightened expression of high-mobility group protein A2 (HMGA2) is associated with human and murine pituitary adenomas [26], and HMGA2 is over-expressed in the pituitary tumors of the TG mice used in this study [8]. HMGA proteins may be involved in the up-regulation of PIT1, a transcription factor implicated in pituitary tumorigenesis [27]. Galanin, a neuropeptide present in the secretory granules in cells of the anterior pituitary and a marker of estrogen status [28], has also been previously shown to be highly up-regulated in βhCG transgenic mice [8]. BMP4 is up-modulated in pituitary adenomas in both mice and humans and acts via SMAD4 to up-regulate c-myc, with signalling mechanisms overlapping with the estrogen receptor [29]. Growth factors like GH and PTTG1 have also been previously shown to be up-regulated in adenomatous pituitaries. PTTG1 participates in tumorigenesis by inducing cell proliferation, transformation and aneuploidy, and has also been shown to induce VEGF [30] and is in turn induced by E2F1 [31]. In TG mice, transcriptional enhancement of several of the genes described above was observed and anti-hCG immunization effectively prevented these increases. The deficiency of the CDK inhibitors CDKN1B and CDKN2C has been associated with the presence of pituitary adenomas in murine models; CDKN1B and CDKN2C possibly control the function of Rb, working collaboratively to suppress pituitary tumorigenesis [32] and CDKN2C is frequently targeted by genomic alterations in pituitary adenomas [33]. As anticipated, transcript levels of CDKN1B, CDKN2A and CDKN2C were greatly lowered in TG mice in the present study; anti-hCG immunization restored levels to those in wild-type mice.

Angiogenesis and vascular remodelling are important processes in embryo implantation and in the development of the placenta [46]. Tumors exploit analogous mechanisms for dissemination, and newer therapeutics seek to target basic fibroblast growth factor (bFGF), vascular endothelial growth factor (VEGF) and platelet derived growth factor (PDGF) [18]. In addition, IL-8 is increasingly recognized for its role in the progression and pathogenesis of cancer [19]. IL-8 and VEGF promote tumor angiogenesis, growth and metastasis, and can be co-expressed in human cancer cells [20]. Neo-angiogenesis and the production of VEGF and IL-8 (both by tumor cells and by tumor-associated macrophages) are accompanied by the heightened secretion of MMPs which aid in endothelial cell and tumor cell escape [21], [22]; in many tumors, MMP-2 and MMP-9 are considered the principal enzymes in this process [23], [24].

Emerging evidence suggests that hCG may exhibit pro-angiogenic effects. Tumor cell-derived hCG was demonstrated to promote sprouting angiogenesis *in vitro* [14] and hCG has been shown to induce the production of IL-8 by monocytes, probably via primitive C-type lectins [47]. Further, hCG has been shown to increase the secretion of MMP-2 and MMP-9 from cytotrophoblastic cells [15]. In the present study, sera derived from TG animals (but not from WT animals) up-modulated the transcription and expression of VEGF, IL-8 and MMP-9 from tumor cells, furthering the premise that hCG (and/or free βhCG) may aid in oncogeneic processes by inducing the production of pro-angiogenic moieties and matrix-degrading enzymes. Active immunization against hCG significantly reduced the capacity of TG sera to induce enhancement in these prominent tumor-promoting factors. In most (but not all) instances, exogenous addition of anti-hCG antibodies to TG sera also reduced transcript and protein levels of the three molecules to near base-line, indicating that βhCG/hCG played a prominent (but perhaps not exclusive) role in their elicitation. Whether the three factors are independently elicited is at present unclear, as are the precise signalling events. It is quite possible that βhCG/hCG induces the generation of a single molecule which in turn leads to the generation of the others via autocrine loops. Indeed, while both IL-8 and VEGF induce the production of MMP-2 and MMP-9 [48], [49], IL8 up-regulates VEGF [50] and VEGF has been shown to enhance IL-8 levels [51]. Whether such a scenario is at work here is under investigation.

Fertility studies, carried out as an assessment of the ability of anti-hCG immunization to restore normalcy to the reproductive axis in transgenic animals, revealed an almost total regain of reproductive performance; the prevention of degenerative changes in the ovaries and pituitaries upon immunization was accompanied by regain of estrus cyclicity in a great majority of immunized females, and mating studies confirmed the ability of the animals to conceive and to deliver healthy progeny. Follow-up studies have revealed that non-transgenic male and female progeny that were born as a result of these matings were also fertile (data not shown).

Given that fact the ectopic expression of hCG is observed in an increasing number of cancers and that its presence is frequently associated with poor patient prognosis, this study provides critical evidence for the potential utility and therapeutic benefits of anti-hCG vaccination in a unique “self” system. Using a variety of biological and molecular readouts, the data demonstrate the utility of immunization protocols capable of breaking tolerance as well as raising antibody titres to levels sufficient to neutralize circulating hormone. These results strengthen the premise that anti-gonadotropin vaccination strategies can prove beneficial in the treatment of malignancy.

## Supporting Information

Table S1 **RT-PCR analysis.** Primer sequences for human and murine actin, and for VEGF, IL-8, MMP9 transcripts.
(DOCX)

Table S2 **RT-PCR analysis.** Primer sequences for murine pituitary transcripts.
(DOCX)

## References

[1] Sheaff MT, Martin JE, Badenoch DF, Baithun SI (1996) Beta hCG as a prognostic marker in adenocarcinoma of the prostate. J Clin Pathol 49: 329–332. 8655711 10.1136/jcp.49.4.329 PMC500461

[2] Crawford RA, Iles RK, Carter PG, Caldwell CJ, Shepherd JH, et al (1998) The prognostic significance of beta human chorionic gonadotropin and its metabolites in women with cervical carcinoma. J Clin Pathol 51: 685–688. 9930074 10.1136/jcp.51.9.685 PMC500907

[3] Lundin M, Nordling S, Carpelan-Holmstrom M, Louhimo J, Alfthan H, et al (2000) A comparison of serum and tissue hCG beta as prognostic markers in colorectal cancer. Anticancer Res 20: 4949–4951. 11326644

[4] Kuroda H, Mandai M, Konishi I, Yura Y, Tsuruta Y, et al (1998) Human chorionic gonadotropin (hCG) inhibits cisplatin-induced apoptosis in ovarian cancer cells: possible role of up-regulation of insulin-like growth factor-1 by hCG. Int J Cancer 76: 571–578. 9590136 10.1002/(sici)1097-0215(19980518)76:4<571::aid-ijc21>3.0.co;2-9

[5] Szturmowicz M, Slodkowska J, Zych J, Rudzinski P, Sakowicz A, et al (1999) Frequency and clinical significance of beta-subunit human chorionic gonadotropin expression in non-small cell lung cancer patients. Tumour Biol 20: 99–104. 10050108 10.1159/000030052

[6] Rulli SB, Kuorelahti A, Karaer O, Pelliniemi LJ, Poutenen M, et al (2002) Reproductive disturbances, pituitary lactotrope adenomas, and mammary gland tumors in transgenic female mice producing high levels of human chorionic gonadotropin. Endocrinology 143: 4084–4095. 12239120 10.1210/en.2002-220490

[7] Huhtaniemi I, Rulli S, Ahtiainen P, Poutanen M (2005) Multiple sites of tumorigenesis in transgenic mice overproducing hCG. Mol Cell Endocrinol 234: 117–126. 15836960 10.1016/j.mce.2004.10.013

[8] Ahtiainen P, Sharp V, Rulli SB, Rivero A, Mamaeva V, et al (2010) Enhanced LH action in transgenic female mice expressing hCGβ-subunit induces pituitary prolactinomas; the role of high progesterone levels. Endocr Relat Cancer 17: 611–621. 20453081 10.1677/ERC-10-0016 PMC2881531

[9] Talwar GP, Singh O, Pal R, Chatterjee N, Sahai P, et al (1994) A vaccine that prevents pregnancy in women. Proc Natl Acad Sci U S A 91: 8532–8536. 8078917 10.1073/pnas.91.18.8532 PMC44640

[10] Moulton HM, Yoshihara PH, Mason DH, Iversen PL, Triozzi PL (2002) Active specific immunotherapy with a beta-human chorionic gonadotropin peptide vaccine in patients with metastatic colorectal cancer: antibody response is associated with improved survival. Clin Cancer Res 8: 2044–2051. 12114402

[11] Gillott DJ, Iles RK, Chard T (1996) The effects of beta-human chorionic gonadotropin on the *in vitro* growth of bladder cancer cell lines. Br J Cancer 73: 323–326. 8562337 10.1038/bjc.1996.56 PMC2074420

[12] Devi GR, Oldenkamp JR, London CA, Iversen PL (2002) Inhibition of human chorionic gonadotropin beta-subunit modulate the mitogenic effect of c-myc in human prostate cancer cells. Prostate 53: 200–210. 12386920 10.1002/pros.10151

[13] Jankowska A, Gunderson SI, Andrusiewicz M, Burczynska B, Szczerba A, et al (2008) Reduction of human chorionic gonadotropin beta subunit expression by modified U1 snRNA caused apoptosis in cervical cancer cells. Mol Cancer 7: 26–34. 18339208 10.1186/1476-4598-7-26 PMC2335103

[14] Zygmunt M, Herr F, Keller-Schoenwetter S, Kunzi-Rapp K, Munstedt K, et al (2002) Characterization of human chorionic gonadotropin as a novel angiogenic factor. J Clin Endocrinol Metab 87: 5290–5296. 12414904 10.1210/jc.2002-020642

[15] Fluhr H, Bischof D, Krenzer BS, Licht P, Bischof P, et al (2008) Human chorionic gonadotropin stimulates matrix metalloproteinases-2 and -9 in cytotrophoblastic cells and decreases tissue inhibitor of metalloproteinases-1, -2 and -3 in decidualized endometrial stromal cells. Fertil Steril 90: 1390–1395. 18291374 10.1016/j.fertnstert.2007.08.023

[16] Singh O, Rao LV, Gaur A, Sharma NC, Alam A, et al (1989) Antibody response and characteristics of antibodies in women immunized with three contraceptive vaccines inducing antibodies against human chorionic gonadotropin. Fertil Steril 52: 739–744. 2806615

[17] Pal R, Singh O, Rao LV, Talwar GP (1990) Bioneutralization capacity of the antibodies generated in women by the beta subunit of human chorionic gonadotropin (beta hCG) and beta hCG associated with the alpha subunit of ovine luteinizing hormone linked to carriers. Am J Reprod Immunol 22: 124–126. 1695850 10.1111/j.1600-0897.1990.tb00654.x

[18] Ballas MS, Chachoua A (2011) Rationale for targeting VEGF, FGF and PDGF for the treatment of NSCLC. Onco Targets Ther 4: 43–58. 21691577 10.2147/OTT.S18155 PMC3116793

[19] Waugh DJ, Wilson C (2008) The interleukin-8 pathway in cancer. Clin Cancer Res 14: 6735–6741. 18980965 10.1158/1078-0432.CCR-07-4843

[20] Bancroft CC, Chen Z, Dong G, Sunwoo JB, Yeh N, et al (2001) Co-expression of proangiogenic factors IL-8 and VEGF by human head and neck squamous cell carcinoma involves coactivation by MEK-MAPK and IKK-NK-kappaB signal pathways. Clin Cancer Res 7: 435–442. 11234901

[21] Belotti D, Paganoni P, Manenti L, Garofalo A, Marchini S, et al (2003) Matrix metalloproteinases (MMP9 and MMP2) induce the release of vascular endothelial growth factor (VEGF) by ovarian carcinoma cells: implications for ascites formation. Cancer Res 63: 5224–5229. 14500349

[22] Luca M, Huang S, Gershenwald JE, Singh RK, Reich R, et al (1997) Expression of interleukin-8 by human melanoma cells up-regulates MMP-2 activity and increases tumor growth and metastasis. Am J Pathol 151: 1105–1113. 9327744 PMC1858026

[23] Masson V, de la Ballina LR, Munaut C, Wielockx B, Jost M, et al (2005) Contribution of host MMP-2 and MMP-9 to promote tumor vascularization and invasion of malignant keratinocytes. FASEB J. 19: 234–236. 10.1096/fj.04-2140fje PMC2771171 15550552

[24] Somiari SB, Somiari RI, Heckman CM, Olsen CH, Jordan RM, et al (2006) Circulating MMP2 and MMP9 in breast cancer - Potential role in classification of patients into low risk, high risk, benign disease and breast cancer categories. Int J Cancer. 119: 1403–1411. 10.1002/ijc.21989 16615109

[25] Yamasaki L, Bronson R, Williams BO, Dyson NJ, Harlow E, et al (1998) Loss of E2f1 reduces tumorigenesis and extends the lifespan of Rb 1(+/−) mice. Nat Genet 18: 360–364. 9537419 10.1038/ng0498-360

[26] Fedele M, Visone R, De Martino I, Troncone G, Palmieri D, et al (2006) HMGA2 induces pituitary tumorigenesis by enhancing E2f1 activity. Cancer Cell 9: 459–471. 16766265 10.1016/j.ccr.2006.04.024

[27] Palmieri D, Valentino T, De Martino I, Esposito F, Cappabianca P, et al (2012) PIT1 upregulation by HMGA proteins has a role in pituitary tumorigenesis. Endocr Relat Cancer 19: 123–135. 22199144 10.1530/ERC-11-0135

[28] Hyde JF, Engle MG, Maley BE (1991) Colocalization of galanin and prolactin within secretory granules of anterior pituitary cells in estrogen-treated Fischer 344 rats. Endocrinology 129: 270–276. 1711463 10.1210/endo-129-1-270

[29] Paez-Pereda M, Giacomini D, Refojo D, Nagashima AC, Hopfner U, et al (2003) Involvement of bone morphogenic protein 4 (BMP-4) in pituitary prolactinomas pathogenesis through a Smad/estrogen crosstalk. Proc Natl Acad Sci U S A 100: 1034–1039. 12552124 10.1073/pnas.0237312100 PMC298721

[30] Vlotides G, Eigler T, Melmed S (2007) Pituitary tumor-transforming gene: physiology and implications for tumorigenesis. Endocr Rev 28: 165–186. 17325339 10.1210/er.2006-0042

[31] Zhou C, Wawrowsky K, Bannykh S, Gutman S, Melmed S (2009) E2f1 induces pituitary tumor transforming gene (PTTG1) expression in human pituitary tumors. Mol Endocrinol 23: 2000–2012. 19837943 10.1210/me.2009-0161 PMC2796149

[32] Franklin DS, Godfrey VL, Lee H, Kovalev GI, Schoonhoven R, et al (1998) CDK inhibitors p18 (INK4c) and p27 (Kip1) mediate two separate pathways to collaboratively suppress pituitary tumorigenesis. Genes Dev 12: 2899–2911. 9744866 10.1101/gad.12.18.2899 PMC317173

[33] Kirsch M, Morz M, Pinzer T, Schackert HK, Schackert G (2009) Frequent loss of the CDKN2C (p18INK4c) gene product in pituitary adenomas. Genes Chromosomes Cancer 48: 143–154. 18973139 10.1002/gcc.20621

[34] Choi JH, Wong AS, Huang HF, Leung PC (2007) Gonadotropins and ovarian cancer. Endocr Rev 2007 28: 440–446. 10.1210/er.2006-0036 17463396

[35] Acevedo HF, Hartsock RJ (1996) Metastatic phenotype correlates with high expression of membrane-associated complete beta-human chorionic gonadotropin *in vivo* . Cancer 78: 2388–2399. 8941011 10.1002/(sici)1097-0142(19961201)78:11<2388::aid-cncr18>3.0.co;2-x

[36] Liu G, Duranteau L, Carel JC, Monroe J, Doyle DA, et al (1999) Leydig cell tumors caused by an activating mutation of the gene encoding the luteinising hormone receptor. N Engl J Med 341: 1731–1736. 10580072 10.1056/NEJM199912023412304

[37] Risma KA, Clay CM, Nett TM, Wagner T, Yun J, et al (1995) Targeted overexpression of luteinizing hormone in transgenic mice leads to infertility, polycystic ovaries, and ovarian tumors. Proc Natl Acad Sci U S A. 92: 1322–1326. 10.1073/pnas.92.5.1322 PMC42511 7877975

[38] Mikola M, Kero J, Nilson JH, Keri RA, Poutanen M, et al (2003) High levels of luteinizing hormone analog stimulate gonadal and adrenal tumorigenesis in mice transgenic for the mouse inhibin-alpha-subunit promoter/Simian virus 40 T-antigen fusion gene. Oncogene 22: 3269–3278. 12761497 10.1038/sj.onc.1206518

[39] Hamada AL, Nakabayashi K, Sato A, Kiyoshi K, Takamatsu Y, et al (2005) Transfection of antisense chorionic gonadotropin beta gene into choriocarcinoma cells suppresses the cell proliferation and induces apoptosis. J Clin Endocrinol Metab 90: 4873–4879. 15886246 10.1210/jc.2004-2458

[40] Cole LA, Khanlian SA, Riley JM, Butler SA (2006) Hyperglycosylated hCG in gestational implantation and in choriocarcinoma and testicular germ cell malignancy tumorigenesis. J Reprod Med 51: 919–929. 17165440

[41] Kumar S, Talwar GP, Biswas DK (1992) Necrosis and inhibition of growth of human lung tumor by anti-alpha-human chorionic gonadotropin antibody. J Natl Cancer Inst 84: 42–47. 1371163 10.1093/jnci/84.1.42

[42] Yankai Z, Rong Y, Yi H, Wentao L, Rongyue C, et al (2006) Ten tandem repeats of β-hCG 109–118 enhance immunogenicity and anti-tumor effects of β-hCG C-terminal peptide carried by mycobacterial heat-shock protein HSP65. Biochem Biophys Res Commun 345: 1365–1371. 16725110 10.1016/j.bbrc.2006.05.022

[43] Freemark M, Fleenor D, Driscoll P, Binart M, Kelly P (2001) Body weight and fat deposition in prolactin receptor deficient mice. Endocrinology 142: 532–537. 11159821 10.1210/endo.142.2.7979

[44] Ratner LD, Gonzalez B, Ahtiainen P, Di Giorgio NP, Poutanen M, et al.. (2012) Short-term pharmacological suppression of the hyperprolactinemia of infertile hCG-overproducing female mice persistently restores their fertility. Endocrinology 153: In press. 10.1210/en.2012-1393 PMC3544356 23117930

[45] Freeman ME, Kanyicska B, Lerant A, Nagy G (2000) Prolactin: structure, function, and regulation of secretion. Physiol Rev 80: 1523–1631. 11015620 10.1152/physrev.2000.80.4.1523

[46] Torry DS, Leavenworth J, Chang M, Maheshwari V, Groesch K, et al (2007) Angiogenesis in implantation. J Assist Reprod Genet 24: 303–315. 17616801 10.1007/s10815-007-9152-7 PMC3455012

[47] Kosaka K, Fujiwara H, Tatsumi K, Yoshioka S, Sato Y, et al (2002) Human chorionic gonadotropin (HCG) activates monocytes to produce interleukin-8 via a different pathway from luteinizing hormone/HCG receptor system. J Clin Endocrinol Metab 87: 5199–5208. 12414893 10.1210/jc.2002-020341

[48] Li A, Dubey S, Varney ML, Dave BJ, Singh RK (2003) IL-8 directly enhanced endothelial cell survival, proliferation, and matrix metalloproteinases production and regulated angiogenesis. J Immunol 170: 3369–3376. 12626597 10.4049/jimmunol.170.6.3369

[49] Ghosh S, Basu M, Roy SS (2012) ETS-1 protein regulates vascular endothelial growth factor-induced matrix metalloproteinase-9 and matrix metalloproteinase-13 expression in human ovarian carcinoma cell line SKOV-3. J Biol Chem 287: 15001–15015. 22270366 10.1074/jbc.M111.284034 PMC3340257

[50] Martin D, Galisteo R, Gutkind JS (2009) CXCL8/IL8 stimulates vascular endothelial growth factor (VEGF) expression and the autocrine activation of VEGFR2 in endothelial cells by activating NFkappaB through the CBM (Carma3/Bcl10/Malt1) complex. J Biol Chem 284: 6038–6042. 19112107 10.1074/jbc.C800207200 PMC2649103

[51] Lee TH, Avraham H, Lee SH, Avraham S (2002) Vascular endothelial growth factor modulates neutrophil transendothelial migration via up-regulation of interleukin-8 in human brain microvascular endothelial cells. J Biol Chem 277: 10445–10451. 11784713 10.1074/jbc.M107348200

