# Supplementary material for: Transgenesis-Mediated Reproductive Dysfunction and Tumorigenesis: Effects of Immunological Neutralization
Source: PLoS One. 2012 Nov 30;7(11):e51125. doi: 10.1371/journal.pone.0051125 (PMC3511405; doi:10.1371/journal.pone.0051125)
Supplement: Table S1 — RT-PCR analysis. Primer sequences for human and murine actin, and for VEGF, IL-8, MMP9 transcripts. (DOCX) [file pone.0051125.s001.docx]

**Table S1.**

| Gene | Forward primer (5’-3’) | Reverse primer (5’-3’) |
| --- | --- | --- |
| Murine ACTB | ATCCGTAAAGACCTCTATGC | AACGCAGCTCAGTAACAGTC |
| Murine VEGFA | CTGTGCAGGCTGCTGTAACG | GTTCCCGAAACCCTGAGGAG |
| Murine IL-8 (KC) | CTTGAAGGTGTTGCCCTCAG | TGGGGACACCTTTTAGCATC |
| Murine MMP-9 | TTGAGTCCGGCAGACAATCC | CCTTATCCACGCGAATGACG |
| Human ACTB | AGATGACCCAGATCATGTTTGAGA | CTAAGTCATAGTCCGCCTAGAAGC |
| Human VEGFA | CCATGAACTTTCTGCTGTCTT | ATCGCATCAGGGGCACACAAG |
| Human IL-8 | AACTTTCAGAGACAGCAGAG | TACAACAGACCCACACAATA |
| Human MMP-9 | CACTGTCCACCCCTCAGAGC | GCCACTTGTCGGCGATAAGG |

ACTB: β-actin; VEGFA: vascular endothelial growth factor A; IL-8: interleukin 8; MMP-9: matrix metalloproteinase 9.
