# Supplementary material for: Transgenesis-Mediated Reproductive Dysfunction and Tumorigenesis: Effects of Immunological Neutralization
Source: PLoS One. 2012 Nov 30;7(11):e51125. doi: 10.1371/journal.pone.0051125 (PMC3511405; doi:10.1371/journal.pone.0051125)
Supplement: Table S2 — RT-PCR analysis. Primer sequences for murine pituitary transcripts. (DOCX) [file pone.0051125.s002.docx]

**Table S2.**

| Gene | Forward primer (5’-3’) | Reverse primer (5’-3’) |
| --- | --- | --- |
| CCND1 | TCTCTTGCTACCGCACAAC | TTCCTCCACTTCCCCCTC |
| HMGA2 | ACCCAGAGGAAGACCCAAAG | CAGTCTCCTGAGCAGGCTTC |
| E2F1 | ACTGTGACTTTGGGGACCTG | CAGAGGGTATGGATCGTGCT |
| PRL | CTCAGGCCATCTTGGAGAAG | TCGGAGAGAAGTCTGGCAGT |
| GAL | GTGACCCTGTCAGCCACTCT | GGTCTCCTTTCCTCCACCTC |
| PTTG1 | AGTTGCCGAAAAGCCTATGA | CCATTCAAGGGGAGAAGTGA |
| BMP4 | CTTCTACAGATGTTTGGGCT | GATGTTCTCCAGATGTTCTT |
| GH | TCCTGTGGACAGATCACTGC | AATGTAGGCACGCTCGAACT |
| CDKN1B | AGGGCCAACAGAACAGAAGA | CCAGATGGGGTGTCAGTTTT |
| CDKN2A | CTTTGTGTACCGCTGGGAAC | CTGAGGCCGGATTTAGCTCT |
| CDKN2C | AATGGATTTGGGAGAACTGC | TGACAGCAAAACCAGTTCCA |
| ACTB | ATCCGTAAAGACCTCTATGC | AACGCAGCTCAGTAACAGTC |

CCDN1: cyclin D1; Hmga2: high mobility group AT-hook 2; E2F1: E2F transcription factor 1; PRL: Prolactin; GAL: Galanin; PTTG1: Pituitary tumor transforming gene 1; BMP4: Bone morphogenetic protein 4; GH: Growth hormone; CDKN1B: Cyclin-dependent kinase inhibitor 1b; CDKN2A: Cyclin-dependent kinase inhibitor 2a; CDKN2C: Cyclin-dependent kinase inhibitor 2c; ACTB: β-actin.
